# Rural community perceptions and practices toward the novel coronavirus (COVID-19) prevention in Konso Zone, Southern Ethiopia: a lesson for the next pandemic

**DOI:** 10.3389/fpubh.2024.1298810

**Published:** 2024-02-07

**Authors:** Gedeno Karbana, Argaw Ambelu, Wondwossen Birke, Lechisa Asefa, Hailu Lemma, Mekdes Mekonen Belay

**Affiliations:** ^1^Department of Environmental Health, Institute of Health, Bule Hora University, Bule Hora, Ethiopia; ^2^Water and Health Division, Ethiopian Institute of Water Resources, Addis Ababa University, Addis Ababa, Ethiopia; ^3^Department of Environmental Health Science and Technology, Public Health Faculty, Institute of Health, Jimma University, Jimma, Ethiopia; ^4^Department of Public Health, College of Medicine and Health Science, Werabe University, Werabe, Ethiopia

**Keywords:** COVID-19, Konso, perception, practice, rural community, Ethiopia

## Abstract

**Background:**

Corona Virus Disease (COVID-19) has provided a lesson on preparedness and coping mechanisms for similar pandemics to different community segments. To control and prevent the spreading of COVID-19, people need to possess the correct attitude and perception to follow the appropriate practices against the causative agent. Therefore, this study aimed to assess the rural community's perceptions and practices toward COVID-19 prevention among southern Ethiopia's Konso zone rural communities.

**Methods:**

A community-based cross-sectional study conducted from April to May 2022 on 605 study participants in the Kena district of the Konso zone of Ethiopia. Participants were recruited using simple random sampling techniques. Face-to-face interviews were conducted to collect data using structured questionnaires developed from the World Health Organization guide and related literature. A logistic regression model was used to identify determinants of perception and practice.

**Results:**

Among the 564 participants, 296 (52.5%) believed they would go to the healthcare facility if they contracted COVID-19. About 276 (48.9%) perceived that COVID-19 is not a stigma and should not be concealed. About 233 (41.3%) perceived COVID-19 would be controlled. However, the majority, 451 (80%), 440 (78%), 384 (68.1%), 381 (67.6%), 337 (59.8%), and 317 (56.2%) perceived that the cause of COVID-19 is sin, eating some food items were effective against the virus, no cases in their locality, living far away from COVID-19 area, the virus does not exist, respectively. Overall, only 22.5% of the study participants have good perceptions. About 58.5% practiced handwashing to prevent COVID-19, and 45.2% covered their mouth and nose while coughing/sneezing. Among the participants, 39.8% were vaccinated, and about a quarter (24.8%) of the respondents had good prevention practices. Participants with better educational status, use of social media as a source of information, and those with better income levels are found to be protective factors while being married is a risk factor.

**Conclusion:**

The status of the participant's perception and preventive practices toward COVID-19 was meager. There was a substantial magnitude of wrong perceptions about controlling such pandemics. High inaccurate perceptions and low preventive practice need an urgent and sustained improvement strategy to tackle similar pandemics or epidemics.

## 1 Introduction

Infectious diseases have become increasingly prevalent and continue to infect and harm people around the world. Disease outbreaks significantly threaten global public health, as evidenced by recent events (1). The COVID-19 pandemic has caused significant loss of life and livelihoods and disrupted economic stability and people's mobility (2, 3). This has resulted in a severe and acute public health crisis that may persist for some time (4).

The coronavirus is highly contagious and has spread globally at an alarming rate (5). As of August 19, 2022, the World Health Organization had reported over 591.68 million confirmed cases and 6.44 million deaths worldwide, with South Africa, Morocco, and Tunisia being the most affected African countries (6). In Ethiopia, as of August 7, 2022, there were 492,491 confirmed COVID-19 cases and 7,569 deaths, with a mortality rate of 1.5% (7). As there is currently no exact treatment for COVID-19, prevention is the most effective strategy to curb the spread of the pandemic (8).

The main measures recommended globally include vaccination, physical distancing, hand washing, staying at home, and wearing face masks (9). The World Health Organization has also developed guidelines and online training sessions to increase community awareness of pandemic prevention (10). However, some residents have not complied with these health and safety measures, as recommended by the WHO and their respective country's health departments (11).

The knowledge, attitudes, and practices (KAP) that people hold toward the disease play a crucial role in determining their readiness to accept behavioral change measures from health authorities (12). The Ethiopian government has implemented several preventive measures, including declaring a state of emergency for nearly 6 months. However, many Ethiopians overlook these measures, exacerbating the spread of the virus (8). To control and prevent COVID-19 infection and transmission, people must have sufficient knowledge of the virus, a positive attitude, and proper viral prevention methods (11). Unfortunately, misinformation has assisted the virus in spreading rapidly across the globe, revealing division, distrust, inequality, and trade tensions (13, 14). During the 2014 Ebola epidemic, a similar picture of misinformation, lack of awareness about the disease outbreak, and negative attitudes was observed, leading to several dangerous practices being carried out by people trying to stay safe (15, 16).

Reports from developed countries indicated significantly better knowledge of symptoms, high-risk groups, transmission routes, and treatment options than those from developing countries (17). Conversely, reports from India showed that study participants had strong knowledge and appropriate practices about the COVID-19 pandemic, but there was a gap in perception of underlying myths and beliefs (18). Studies conducted in Ethiopia found that cultural and religious dependencies, the belief that the pandemic does not affect the young, misinformation about the disease, and a lack of trust in prevention measures were the major obstacles to practicing COVID-19 prevention measures (19, 20). A study in northwest Ethiopia also showed poor KAP during the pandemic (21). Most studies on the KAP of COVID-19 have focused on urban settings, despite the pandemic's impact on both urban and rural communities. Poor healthcare facilities, limited availability of testing kits, isolation facilities, and low levels of awareness due to a lack of information on preventive measures could make controlling the virus difficult in rural areas (5). For example, the country's capital city, Addis Ababa, reported the highest caseload with 334,202 cases compared to the country's regional states (7). Although much has been done to assess the KAP of COVID-19 worldwide, including in Ethiopia, greater attention has been given to urban communities with internet access through different media platforms than to rural communities, which comprise most of the population.

Understanding local perceptions of COVID-19 among residents and COVID-19 prevention practices in the context of people's socio-cultural and educational backgrounds is crucial in designing educational interventions to promote COVID-19 control measures and strengthen adherence to these measures. Therefore, it is important to assess rural community perceptions and practices of COVID-19 in the Kena district, to fill gaps in perception and practice toward COVID-19 and similar pandemics and to assist the fight against the pandemic in a local context.

## 2 Methods and materials

### 2.1 Study setting and period

From April 21 to May 6, 2022, a study was carried out in the Kena district, which is located in the Konso zone of southern Ethiopia. The research collected information about the COVID-19 pandemic in the area. The district is situated about 574 km from Addis Ababa, the capital city of Ethiopia, and 247 km from Hawassa, the capital city of the South Nation Nationalities and Peoples Region. It encompasses 10 kebeles, which are the lowest level of government administration in Ethiopia, and has a total population of 82,019 with 14,217 households. Additionally, there are four health centers and 17 health posts in the district at the time of the study.

### 2.2 Study design

A community-based cross-sectional study was conducted among rural communities to assess their perceptions and practices toward the pandemic.

### 2.3 Source and study populations

All the people in the Kena district of southern Ethiopia were the source population. All the eligible individuals aged 18 years and older in selected households that belong to the randomly selected three Kebeles of the district were included in the study. Those who were severely ill at the time of data collection were excluded.

### 2.4 Sample size determination

The sample size was determined using the single population proportion formula by considering the following assumptions.


$$n\;=\;\frac{\left(Z_{\alpha_{2}})2^{*}P^{*}(1-P\right)}{d^{2}}$$

Where: *n*: sample size.

*Z*: the standard normal variance at 95% CI (1.96).

*P*: the proportion of good perception and practice level in the local community is taken as 50% since no previous study has been done similar to this; *p* = 0.5; *q* = (1–*p*) = (1–0.5) = 0.5.

*d*: marginal errors 5% = 0.05.


$$n\;=\;\frac{\left(1.96\right)2^{*}0.5^{*}(1-0.5)}{{\left(0.05\right)}^{2}}$$

n = 384

Since simple random sampling techniques were used at Kebele and at the household level to select an eligible study participant in the study area, a 1.5 design effect was accounted for. Then the final sample size with a 5% non-response rate was 605.

### 2.5 Sampling technique and procedures

**Figure 1:**
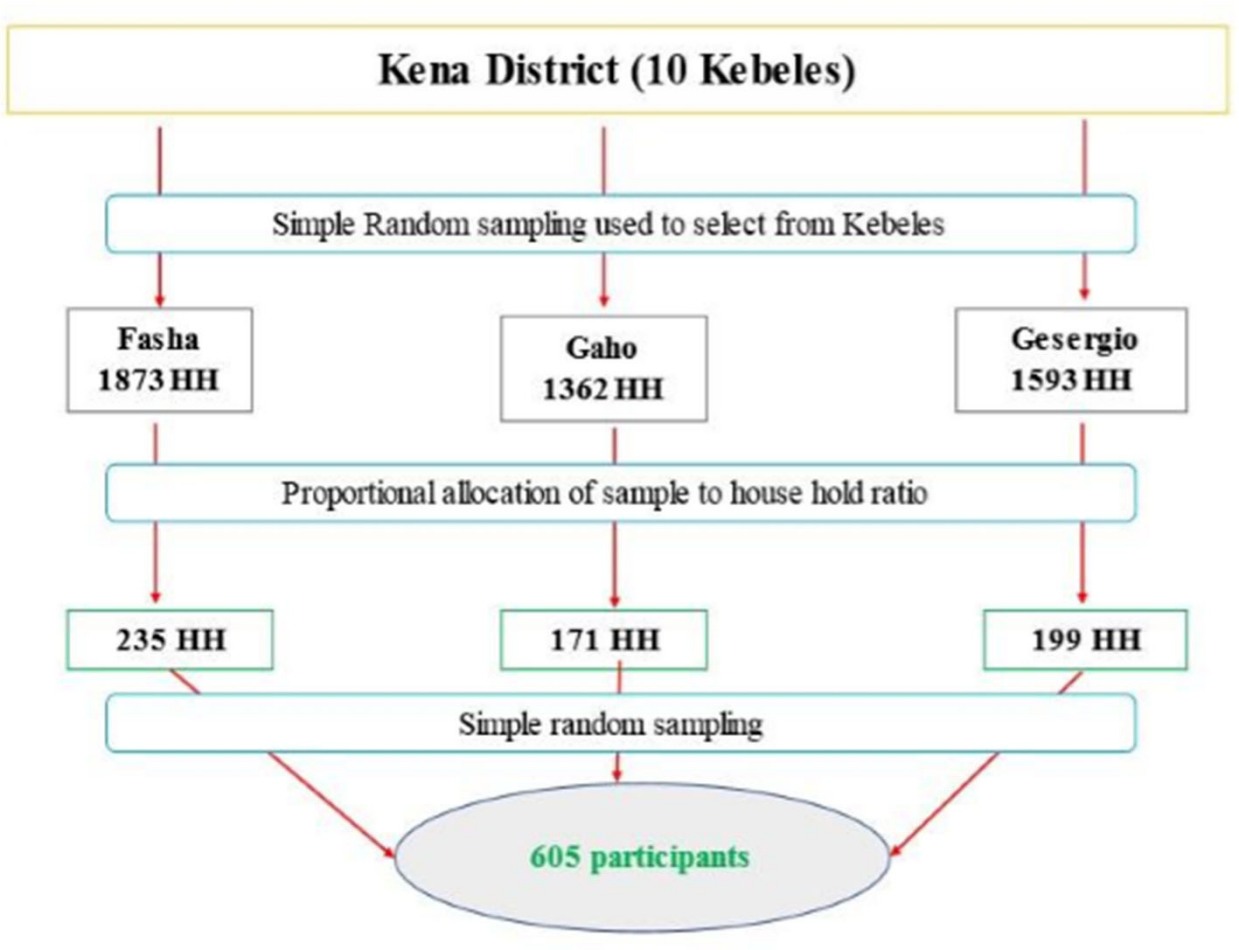
Sampling techniques and procedures used to select study participants in Kena district, Konso zone, southern Ethiopia, 2022.

To establish the sample size, three Kebeles were chosen randomly out of ten, as displayed in Figure 1. The number of sample households was then proportionally allocated to the selected Kebeles. A random number generator was employed to choose households from each Kebele, using the family roster previously registered at the respective health posts. Finally, for each chosen household, individuals aged 18 or above were randomly selected through the lottery method in case the household head was not present during data collection.

### 2.6 Study variables

The perceptions and practices of the respondents toward COVID-19 were the dependent variables. Whereas, sociodemographic characteristics like age, sex, marital status, educational status, occupation, family size, monthly income, and source of information were the independent variables.

### 2.7 Data collection, management, and quality assurance

We gathered the data through a structured questionnaire, which we adapted in English from the WHO Risk Communication and Community Engagement (RCCE) action plan guidance and various related literature (8, 20, 22, 23). Next, we translated the questionnaire into Amharic to ensure that the data collectors and interviewees could better understand it. The questionnaire included questions about demographics, perceptions, preventative practices, and sources of information. Before beginning the actual data collection, we pretested the questionnaire on 30 households outside the selected kebeles in the Karat Zuria district using the Amharic version to make any necessary amendments. Experienced supervisors provided training and instructions to the data collectors.

### 2.8 Statistical analysis

First, the data collected was coded and entered into Epi-Data version 3.1. It was then exported to SPSS version 23 for analysis after cleaning it. Before the final analysis, all variables were recoded and transformed as needed. The variables were described using frequency distributions, cross-tabulations, and graphs. After evaluating the normal distribution of the data, bivariate logistic regression was used to analyze the association between the outcome and predictor variables. Each independent variable was analyzed separately, and predictor variables with a *p*-value of <0.25 were selected for the final model. All candidate variables were then analyzed using multivariate logistic regression. The Hosmer-Lemeshow test was used to check the model's fitness. An adjusted odds ratio with a 95% confidence interval was calculated, and *P*-values <0.05 were considered statistically significant. Finally, the results were presented using texts, graphs, figures, and tables.

### 2.9 Contextual definitions

**The perception:** Was dichotomized as good if the overall score was > 50% and poor if ≤ 50% (24). It consisted of 14 questions, and participants who correctly answered more than seven questions were categorized as “good perception,” and those who scored less than or equal to seven were categorized as “poor perception.”

**The practice:** Was dichotomized as good if the overall score was ≥ 50% and poor if <50% (24, 25). The practice questionnaire consisted of seven questions, and participants who correctly answered four or more questions were categorized as “good practice,” and those who scored <4 were categorized as “poor practice.”

**Inaccurate beliefs:** Misperception resulting from incorrect reasoning. The unscientific way the rural community thinks about how to prevent and how they interpret information related to the current global COVID-19 pandemic.

**Correct perception:** The scientific way the rural community thinks about how to prevent and how they interpret information related to the current global pandemic COVID-19.

### 2.10 Ethical considerations

The Ethical Committee of Jimma University has approved the study protocol for collecting the data from the respondents. IRB letter reference number is IHRPG1/442/22. The respondent's right to refuse or withdraw from participating was fully maintained, and the information provided by each respondent was kept strictly confidential. Written consent was obtained from each of the study participants.

## 3 Results

### 3.1 Sociodemographic characteristics of the respondents

**Figure 2:**
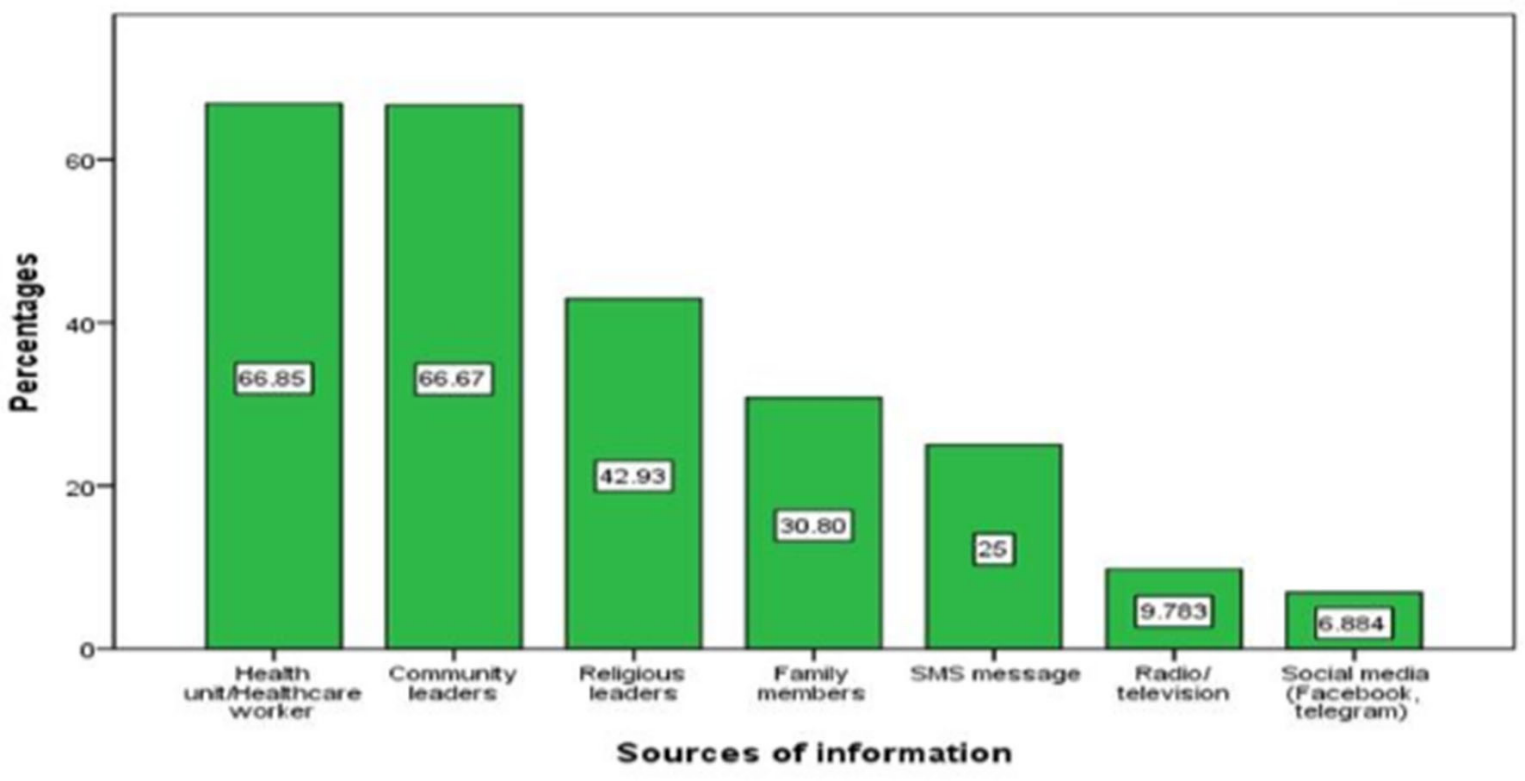
Source of information about the global pandemic in Kena district, southern Ethiopia, April–May 2022 (n = 564).

This study had 583 participants, with a response rate of 96.4%. Of the total participants, 304 (53.9%) were female. The majority, 453 (80.5%) were married and 244 (43.3%) were housewives (Table 1). Refer to Figure 2 for the source of information the respondents had heard about the pandemic. Out of the 583 respondents, 564 (96.74%) claimed that they had heard about COVID-19.

**Table 1:** Sociodemographic characteristics of the respondents in Kena district, southern Ethiopia, April–May 2022.

**Variables**	**Response category**	**Frequency**	**Percentage**
Sex	Male	260	46.1
	Female	304	53.9
Age in years	18–29	177	31.4
	30–39	227	40.2
	≥40	160	28.4
Educational level	Cannot read and write	279	49.5
	No formal education but read and write	73	12.9
	Primary (1–8 grade)	111	19.7
	Secondary (9–12 grade)	46	8.2
	College and above	55	9.8
Marital status	Single	88	15.6
	Married	454	80.5
	Other	22	3.9
Occupational status	Farmer	167	29.6
	Merchant	49	8.7
	Housewife	244	43.3
	Employed	32	5.7
	Student	55	9.8
	Other	17	3.0
Religion	Protestant	403	71.5
	Orthodox	154	27.3
	Other	7	1.2
Family size	≤ 4	160	28.4
	>4	404	71.6
Monthly Income (ETB)	<1,000	130	23.0
	1,000–1,999	270	47.9
	2,000–4,000	136	24.1
	>4,000	28	5.0

n = 564.

ETB, Ethiopian Birr.

### 3.2 Perception and practice of respondents toward the COVID-19 disease

**Figure 3:**
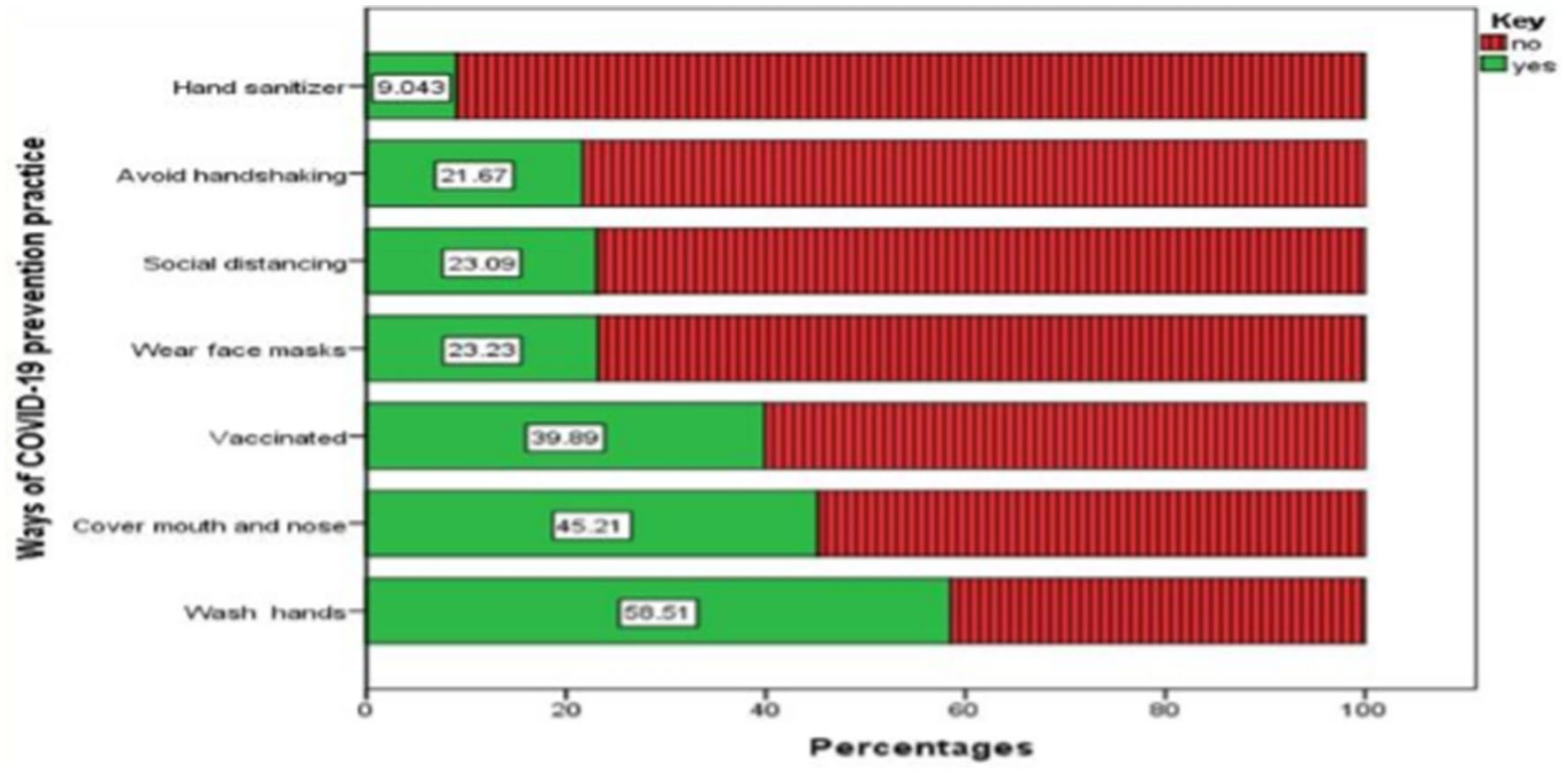
The status of the prevention practice of the participants toward COVID-19 among the residents of Kena district, southern Ethiopia, April–May 2022 (n = 564).

**Figure 4:**
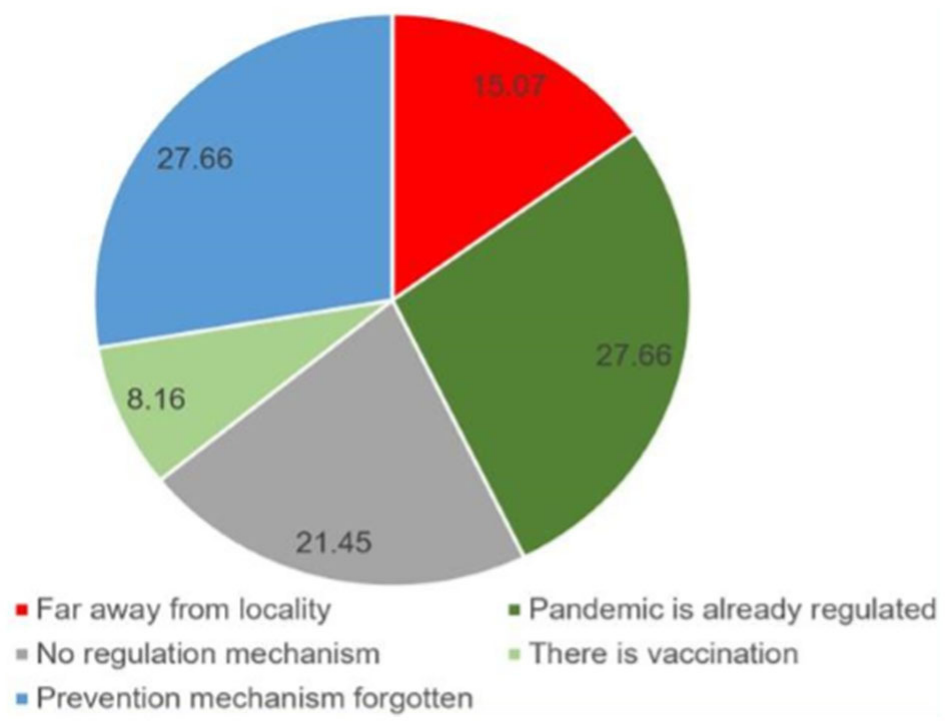
Responses of the participants for not practicing COVID-19 prevention practices in Kena district, southern Ethiopia, April–May 2022 (*n* = 564).

In Table 2, the study participants' perceptions of the pandemic are presented. More than half of the participants (52.5%) indicated that they would seek medical attention if they were infected. However, the majority of respondents (80%) mistakenly believed that COVID-19 was caused by sin, and a significant portion (78%) believed that using certain food items and spices like garlic, ginger, honey, and lemon as home remedies could effectively combat the virus. Figure 3, a graph below, displays the prevention practices of the rural community participants. Shockingly, a vast majority of them (91%) did not use hand sanitizer when soap and water were not available, while 78.3% did not avoid handshakes when greeting others. In contrast, Figure 4 illustrates the reasons why some participants were not practicing prevention methods.

**Table 2:** Perception toward COVID-19 disease in Kena district, southern Ethiopia, April–May 2022 (*n* = 564).

**Perception of respondents toward COVID-19**	**Freq. (%)**	
	**Yes**	**No**
Do you think you can contract COVID-19?	199 (35.3)	365 (64.7)
If you were getting infected with COVID-19, would you go to the health facility?	296 (52.5)	268 (47.5)
Do you think you are well-informed about the current pandemic?	228 (40.4)	336 (59.6)
Do you think COVID-19 is not a stigma and you should not hide the infection?	276 (48.9)	288 (51.1)
Would you think COVID-19 will be successfully controlled?	233 (41.3)	331 (58.7)
Do you believe using garlic, ginger, etc. as home remedies are a necessity to confront coronavirus?^*^	124 (22.0)	440 (78.0)
Do you believe drinking local alcohol is necessary to protect against COVID-19?^*^	315 (55.9)	249 (44.1)
Do you think you are living far away from COVID-19′s rampant areas?^*^	183 (32.4)	381 (67.6)
Do you think there are no locally reported COVID-19 cases so far?^*^	180 (31.9)	384 (68.1)
Do you believe you are religious enough to control COVID-19?^*^	222 (39.4)	342 (60.6)
Do you believe you have traditional medicine against COVID-19?^*^	247 (43.8)	317 (56.2)
Do you think that the cause of COVID-19 is happened because of our sins?^*^	113 (20.0)	451 (80.0)
You don't believe COVID-19 exists?^*^	227 (40.2)	337 (59.8)
Do you think the disease is being exaggerated?^*^	303 (53.7)	261 (46.3)

^*^Correction rate calculated from “No” response for false statements.

### 3.3 Factors associated with the status of perception on COVID-19

Our analysis used binary logistic regression to identify factors associated with respondents' perceived status of the pandemic. The model showed that sex, age, educational level, marital status, occupational status, income level, and various sources of information such as healthcare workers, radio/television, religious leaders, SMS messages, and social media were significant. Additionally, practice level was also found to be significant. All of these variables were included in the multivariable analysis model, as shown in Table 3.

**Table 3:** Factors determining the level of perception toward COVID-19 among Kena district residents, southern Ethiopia, April–May 2022 (*n* = 564).

**Variables**	**Response category**	**Perception level**		**COR (75% CI)**	***p*-value**	**AOR (95% CI)**	***p*-value**
		**Poor (%)**	**Good (%)**				
Educational level	Cannot read and write	246 (88.2)	33 (11.8)	1		1	
	No formal education but read and write	60 (82.2)	13 (17.8)	1.61 (1.07–2.43)	0.180^*^	1.26 (0.56–2.82)	0.566
	Primary (1–8 grade)	92 (82.9)	19 (17.1)	1.54 (1.07–2.20)	0.168^*^	0.95 (0.43–2.12)	0.913
	Secondary (9–12 grade)	24 (52.2)	22 (47.8)	6.83 (4.57–10.20)	0.0001^*^	4.05 (1.32–12.36)	**0.014** ^**^
	College and above	15 (27.3)	40 (72.7)	19.87 (13.21–29.90)	0.0001^*^	4.71 (1.19–18.63)	**0.027** ^**^
Source of information using social media (FB, telegram)	Yes	9 (23.7)	29 (76.3)	14.07 (8.9–22.23)	0.0001^*^	3.69 (1.31–10.39)	**0.013** ^**^
	No	428 (81.4)	98 (18.6)	1		1	

COR, Crude Odd Ratio; AOR, Adjusted Odd Ratio; ETB, Ethiopian Birr.

^*^p-value <0.25 statistically significant, ^**^p-value <0.05 statistically significant. Bold values indicate that their *p*-values are <0.05.

After analyzing multiple factors using a multivariate analysis, it was found that a person's level of education and their use of social media as a source of information has a significant impact on their perception. Individuals with secondary education were found to be four times more likely to have a good perception compared to those who cannot read or write. Similarly, college students and those with higher education were 4.71 times more likely to have a good perception than their counterparts. Additionally, those who use social media as a source of information were found to be three times more likely to have a good perception compared to those who don't use social media.

### 3.4 Factors associated with the practice of COVID-19 prevention

To know the associated factors with the status of the prevention practice, all the variables were entered into the binary logistic regression model (Table 4). Accordingly, sex, age, educational level, marital status, occupational status, income level, SMS message as a source of information, social media as a source of information, and perception level had a *p*-value of <0.25 and fitted into the multivariable analysis model.

**Table 4:** Factors determining COVID-19 prevention practice among Kena district residents, southern Ethiopia, April–May 2022 (*n* = 564).

**Variables**	**Response category**	**Practice level**		**COR (75% CI)**	***p*-value**	**AOR (95%CI)**	***p*-value**
		**Poor (%)**	**Good (%)**				
Educational level	Cannot read and write	237 (84.9)	42 (15.1)	1		1	
	No formal education but read and write	54 (70.0)	19 (26.0)	1.98 (1.38–2.85)	0.029^*^	1.31 (0.65–2.63)	0.436
	Primary (1–8 grade)	72 (64.9)	39 (35.1)	3.05 (2.26–4.12)	0.0001^*^	2.19 (1.14–4.21)	**0.018** ^**^
	Secondary (9–12 grade)	30 (65.2)	16 (34.8)	3.01 (2.00–4.51)	0.002^*^	2.77 (0.96–7.95)	0.059
	College and above	31 (56.4)	24 (43.6)	4.36 (3.02–6.30)	0.0001^*^	2.56 (0.67–9.82)	0.169
Marital status	Single	51 (58.0)	37 (42)	1		1	
	Married	353 (77.8)	101 (22.2)	0.39 (0.29–0.52)	0.0001^*^	0.41 (0.18–0.94)	**0.035** ^**^
	Widowed	20 (90.9)	2 (9.1)	0.13 (0.05–0.33)	0.010^*^	0.23 (0.04–1.32)	0.100
Monthly income (ETB)	<1,000	109 (83.8)	21 (16.2)	1		1	
	1,000–1,999	212 (78.5)	58 (21.5)	1.42 (1.03–1.96)	0.211^*^	1.47 (0.78–2.77)	0.226
	2,000–4,000	91 (66.9)	45 (33.1)	2.57 (1.82–3.63)	0.002^*^	1.95 (0.90–4.22)	0.088
	>4,000	12 (42.9)	16 (57.1)	6.92 (4.12–11.61)	0.0001^*^	3.89 (1.03–14.77)	**0.042** ^**^

COR, Crude Odd Ratio; AOR, Adjusted Odd Ratio; ETB, Ethiopian Birr.

^*^p-value <0.25, ^**^p-value <0.05 statistically significant. Bold values indicate that their *p*-values are <0.05.

In multivariable logistic regression analysis, educational level, marital status, and income level affected the prevention practice. It was revealed that those who attended primary education were two times (AOR = 2.19, 95% CI 1.38–4.88) more likely to use good practices to prevent COVID-19 than those who cannot read and write. The odds of having good preventive practice were four times (AOR: 3.89; 95% CI: 1.03–14.77) among the monthly income level category >4,000 than those who had <1,000 income level. Moreover, being married was 59% times (AOR: 0.41; 95% CI: 0.18–0.94) less likely to have good COVID-19 prevention practices than being single.

## 4 Discussion

This research examined how rural communities perceive and respond to the global pandemic 2 years after its emergence. The study identified factors that influence their perception and practices in preventing the spread of the virus. The results can provide valuable insights for health offices, health extension workers, and other stakeholders in rural areas. It is essential to use these findings to promote behavioral change and maintain preventive measures, as well as address any gaps in current literature.

According to our finding, rural communities primarily relied on healthcare workers (66.8%) and community leaders (66.6%) for information. These communities trusted health workers and community leaders, including health extension workers, the health development army, and health professionals. However, studies conducted in Tigray, Gedeo zone, and Southern Ethiopia (24–26) found that most residents obtained COVID-19 information from TV/radio and social media. This difference may be due to the location and population of the study area. The study participants were from remote areas without telecommunications and electrical services. This highlights the need for tailored interventions to increase awareness about COVID-19 and mitigation measures, utilizing professionals and community leaders.

The study found that a significant number of respondents (ranging from 44.1 to 80%) held inaccurate beliefs about COVID-19. Specifically, they believed that consuming local alcohol, certain food items (such as garlic, ginger, and honey), and traditional medicines could protect them from the virus. Some respondents also subscribed to religious myths, such as the belief that the pandemic was caused by an increase in human sin, and that being religious enough could control the spread of the virus. Additionally, false assurances from local people that they were safe from the virus, as well as the belief that COVID-19 did not exist, contributed to the prevalence of inaccurate beliefs. The study found that this level of inaccurate belief was higher than in a previous study conducted in Ethiopia (20). The study suggests that such inaccurate beliefs are damaging and could hinder efforts to prevent and reduce the spread of COVID-19. It is therefore important for concerned bodies to make more effort to reduce these beliefs and improve education around COVID-19.

On the other hand, in this study, there were lower reports on the correct perception statements, ranging from 35.3 to 52.5% which was found inconsistent with the study done in Northwest Ethiopia and Northern Ethiopia (8, 24). These differences might be related to educational status, cultural beliefs, and study periods. Thus, adequate and sustained health information should be provided to the community. Generally, the current study revealed that the overall perception of the rural community was very poor (22.5%). This finding is lower compared to studies conducted in northeast Ethiopia (57.9%) (27), northwest Ethiopia (62.6%) (8), and southern Ethiopia (90.3%) (28) where their perception level toward COVID-19 is higher. This variation might be due to the study area, period, and population. The current study was done after 2 years of the emergence of the pandemic, unlike previous studies.

Furthermore, this finding was lower than perception studies on COVID-19 from Egypt (29), and Saudi Arabia (30). These discrepancies might be due to the study period, geographical areas, and the number and type of questions used. The previous studies were done at the beginning, but the current one was conducted after 2 years. The former abroad studies' participants have better access to healthcare information compared to this rural community study.

The current study depicted that the status of perception regarding COVID-19 was affected by educational status, and social media as a source of information. Those who had secondary education and above college were four times more likely to have good perception than those who could not read and write. This study was consistent with studies from Northeast Ethiopia (23) and Southern Ethiopia (28) where higher educational level was positively associated with good perception than their reference group. This might be a result of educated individuals being more informed (getting more information through various communication platforms) about the illness and suggested preventative measures.

Furthermore, the current study demonstrated that participants who had exposure to social media as a source of information had a good perception of COVID-19. This is consistent with the previous study (24) which looked if social media could be wisely used might bring behavioral change.

Due to the absence of an exact treatment, prevention is a highly recommended strategy to control the spread of the COVID-19 pandemic (8). As a result, the main measures recommended globally to prevent the pandemic were vaccination, physical distance, hand washing, staying at home, and wearing face masks (9). However, in this study, only a quarter (24.8%) of the respondents practiced the recommended prevention measures. This finding is higher compared to findings from Dirashe district (31) and Gedeo zone (28), Ethiopia, where the preventive practices were 12.3 and 20%, respectively. This variation might be related to changes in the study setting, study period, socio-economic factors, and awareness of the communities.

On the other hand, the practice level of the current study was found to be lower than previous studies done in Ethiopia. It was lower than Addis Ababa (59.8%) (32), and Sidama region (65%) (33) studies conducted on preventive measures. This discrepancy might be explained because of the difference in the study period, the number of study participants, and the cut-off point used to categorize the preventive measures into poor or good status. When the study period would be seen, the previous studies were conducted a few months after the occurrence of the pandemic while the current study was done after 2 years of the emergence of the disease. This indicates prevention measures should be practiced sustainably.

Moreover, the preventive practices in the current study were found lower than the studies done in India (88.1%) (18), and Malaysia (95.9%) (12). This variation might be related to socio-economic status, study period & population, and methods of data collection. In this study, data was collected face-to-face among the most illiterate category of respondents, while the former studies were collected online.

Respondents of this study were also asked about the reason why they didn't practice the recommended preventive measures for the virus. As a result, the study participants' main reason was they perceived that the disease was controlled/regulated and reduced 27.6%, similarly, they considered the issue of the diseases was forgotten even by asking the data collectors back by saying “*Is there incidence/case of COVID-19 present?*” The current reasoning was not in agreement with the study conducted in Dessie town, Northeast Ethiopia which reasoned that it “takes too much effort” (27). This difference may be related to the variation in the study setting, period, participants, and type of questions. Because the current study was conducted after 2 years after the occurrence of the pandemic compared to its counterpart. It might be suggested that the residents were performing the prevention method because of the enforcement of the regulatory bodies but not understanding the risk of the virus. From these responses, the low role-playing of concerned bodies could be understood which might awaken the bodies to sustain the recommended preventive measures and behaviors.

Based on the multivariable model, those who attended primary education were twice more likely to have good practices to prevent COVID-19 than those who cannot read and write. The study conducted in southern Ethiopia (28) supported this association. This shows the higher the educational level, the higher the practice of preventive measures against the disease. Additionally, the odds of having good preventive practice were four times higher among the income level category >4,000 than those who had <1,000 income level. This is consistent with the study conducted in the Sidama region in Ethiopia (33). This shows that those who have higher incomes can afford the recommended preventive personal protective equipment, like face masks, hand sanitizers, and soap. Furthermore, marital status was found to be statistically associated with the prevention practices of the participants. Participants who had married were 59% times less likely to practice the prevention measures for COVID-19 than those who didn't (single ones). The current study differs from the study done in the Sidama region, Ethiopia (33), where those married were twice as practicing the prevention measures than their reference group. This variation might be because of sociocultural disparities, levels of knowledge, access to information sources, etc.

In Ethiopia, around 80% of the citizens live in rural areas. The majority of the population residing in these rural areas face a variety of health-related problems due to poor access to transportation, poor healthcare facilities, lack of awareness, and a shortage of well-equipped facilities in the local area. Similarly, COVID-19 awareness creation activities and preventive practices did not reach these remote areas. In addition, studies conducted in Ethiopia on COVID-19 had given greater attention to urban communities with internet access through different media platforms like social media, mass media, and telecom. However, all these channels are not accessible and suitable for rural communities. Previous studies are also focused on educated population (internet users). Hence, strengthening existing local health systems like health extension packages, and health development armies and continuously improving the perception of the local communities is imperative. Therefore, scholars must reach this remote unprivileged population to develop appropriate intervention measures based on the local context.

Having strengths, this study may face limitations like assessment of the cause–effect relationship was not possible due to the cross-sectional nature of the study, there could be social desirability, and the knowledge of community members on the illness was not addressed.

## 5 Conclusion

Finally, the study revealed that a high number of the study participants had poor perceptions and prevention practices toward the control of the pandemic in the community. There was a substantial magnitude of wrong perceptions and very low. Educational level, income level, knowledge status, and social media as a source of information acted as protective factors at a *p*-value of 0.05 against the prevention of the pandemic. While marital status was a risk factor for COVID-19 prevention. Therefore, the risk communication and community engagement efforts in collaboration with concerned bodies should investigate the beliefs that could exacerbate/inhibit the spread of the disease, provide the communities with real information about the virus, and work on prevention measures sustainably. Behavioral change communications are also imperative to strengthen COVID-19 prevention practices. It needs an urgent and sustained improvement strategy to tackle similar pandemics or epidemics.

## Data availability statement

The raw data supporting the conclusions of this article will be made available by the authors, without undue reservation.

## Ethics statement

The Ethical Committee of Jimma University has approved the study protocol for collecting the data from the respondents. IRB letter reference number is IHRPG1/442/22. The respondent's right to refuse or withdraw from participating was fully maintained, and the information provided by each respondent was kept strictly confidential. Written consent was obtained from each of the study participants.

## Author contributions

GK: Conceptualization, Formal analysis, Methodology, Writing—original draft. AA: Supervision, Writing—review & editing. WB: Supervision, Writing—review & editing. LA: Writing—review & editing. HL: Writing—review & editing. MB: Writing—review & editing.
