# Supplementary material for: Rural community perceptions and practices toward the novel coronavirus (COVID-19) prevention in Konso Zone, Southern Ethiopia: a lesson for the next pandemic
Source: Front Public Health. 2024 Feb 7;12:1298810. doi: 10.3389/fpubh.2024.1298810 (PMC10879358; doi:10.3389/fpubh.2024.1298810)
Supplement: Supplementary file 1 [file Table_1.DOCX]

**Rural community perceptions and practices towards the novel coronavirus (COVID-19) prevention in Konso Zone, southern Ethiopia: A lesson for the next pandemic**

Gedeno Karbana*^1^, Argaw Ambelu^2^, Wondwossen Birke^3^, Lechisa Asefa^1^, Hailu Lemma^1^ Mekdes Mekonen Belay^4^

^1^Lecturers at Department of Environmental Health, Institute of Health, Bule Hora University, Bule Hora, Ethiopia.

^2^Professor at Water and Health Division, Ethiopian Institute of Water Resources, Addis Ababa University, Addis Ababa, Ethiopia.

^3^Lecturer at Department of Environmental Health Science and Technology, public health faculty, Institute of Health, Jimma University, Jimma, Ethiopia.

^4^Lecturer at Department of Public Health, College of Medicine and Health Science, Werabe University, Werabe, Ethiopia.

*Correspondence

Corresponding Author: Gedeno Karbana

Email: gedeno3472@gmail.com

**A questionnaire to assess rural community perceptions and practices regarding the Novel Coronavirus (COVID-19) prevention in Kena District, Konso Zone, Southern Ethiopia, 2022.**

**Name of the kebele ______________________ Date____________________________**

**Name of specific place __________________ Code ______________________**

| **s.no.** | **Question** | **Responses** |
| --- | --- | --- |
| **1** | **Socio-demographic characteristics of the Study participants** | |
| 1.1 | Sex | a. Male  b. Female |
| 1.2 | Age (in years) | _____________ |
| 1.3 | Educational level | a. Cannot read and write  b. No formal education but read and write  c. Formal education (write highest grade completed) ______ |
| 1.4 | Marital status | a. Single  b. Married  c. Divorced  d. Widowed  e. Separated |
| 1.5 | Occupational status | a. Farmer  b. Merchant c. Employed d. Unemployed e. Student  f. Other (specify)___________ |
| 1.6 | Religion | a. Protestant  b. Orthodox  c. Muslim  d. Other (specify)___________ |
| 1.7 | Number of family size | __________________ |
| 1.8 | Monthly income (ETB) | __________________ |
| **2** | **Source of information about COVID-19** | |
| 2.1 | Have you ever heard about the coronavirus disease (COVID-19)? | a. Yes  b. No |
| 2.2 | If yes, from where did you heard about the coronavirus? What channels or sources? (Multiple answers) | A. Health unit/Health care worker  B. Family members  C. Radio/ television  D. Religious leaders  E. Community leaders  F. SMS message  G. social media (Facebook, telegram) |
| **3** | **Perception of respondents towards the COVID-19 outbreak in Kena rural communities** | |
| 3.1 | Do you think you can contract COVID-19? | a. yes b. no |
| 3.2 | If you were getting infected with COVID-19, would you go to the health facility? | a. yes b. no |
| 3.3 | Do you think you are well informed about the current pandemic? | a. yes b. no |
| 3.4 | COVID-19 is not a stigma and I should not hide my infection. | a. yes b. no |
| 3.5 | would you think COVID-19 will be successfully controlled? | a. yes b. no |
| 3.8 | **Local perceptions toward Covid-19 prevention** | |
|  | a. Do you believe using garlic, ginger, etc. as home remedies are a necessity to confront coronavirus? | a. yes b. no |
|  | b. Do you believe drinking local alcohol is necessary to protect against COVID-19 | a. yes b. no |
|  | c. Do you think you are living far away from COVID-19’s rampant areas | a. yes b. no |
|  | d. Do you think there are no locally reported COVID-19 cases so far? | a. yes b. no |
|  | e. Do you believe you are religious enough to control COVID-19? | a. yes b. no |
|  | f. Do you believe you have traditional medicine against COVID-19? | a. yes b. no |
|  | 1. Do you think that the cause of Covid-19 is happened because of our sin? | a. yes b. no |
|  | 1. You don’t believe COVID-19 exists? | a. yes b. no |
|  | 1. Do you think the disease is being exaggerated? | a. yes b. no |
| **4** | **Practices of respondents towards the COVID-19 outbreak in Kena local communities** | |
| 4.1 | Do you avoid handshaking to prevent covid-19? | a. yes b. no |
| 4.2 | Do you Frequently wash your hands with soap? | a. yes b. no |
| 4.3 | If soap and water are not readily available, do you apply a hand sanitizer? | a. yes b. no |
| 4.4 | Do you cover your mouth and nose while coughing? | a. yes b. no |
| 4.5 | Do you Practice social distancing of at least 1 meter? | a. yes b. no |
| 4.6 | Do you wear face masks repeatedly when you leave your home? | a. yes b. no |
| 4.7 | Have you been vaccinated? | a. yes b. no |
| 4.8 | If you don’t practice reason for not practicing? | 1. Far away from the locality 2. Reduced/regulated 3. No regulation 4. Presence of vaccine 5. forgotten 6. Other________ |
